# Effects of second-line antihyperglycemic drugs on the risk of chronic kidney disease: applying a target trial approach to a hospital-based cohort of Thai patients with type 2 diabetes

**DOI:** 10.1186/s12933-022-01641-2

**Published:** 2022-11-17

**Authors:** Sukanya Siriyotha, Thitiya Lukkunaprasit, Panu Looareesuwan, Hataikarn Nimitphong, Gareth J. McKay, John Attia, Ammarin Thakkinstian

**Affiliations:** 1 Department of Clinical Epidemiology and Biostatistics, Faculty of Medicine Ramathibodi Hospital, Mahidol University, Bangkok, Thailand; 2 Department of Pharmacy Administration, College of Pharmacy, Rangsit University, Pathum Thani, Thailand; 3 Division of Endocrinology and Metabolism, Department of Medicine, Faculty of Medicine Ramathibodi Hospital, Mahidol University, Bangkok, Thailand; 4 Centre for Public Health, School of Medicine, Dentistry and Biomedical Sciences, Queen’s University Belfast, Belfast, UK; 5 Centre for Clinical Epidemiology and Biostatistics, School of Medicine and Public Health, Faculty of Health and Medicine, University of Newcastle, and Hunter Medical Research Institute, New Lambton, NSW Australia

**Keywords:** Antihyperglycemic drugs, Chronic kidney disease, Sodium-glucose cotransporter-2 inhibitors, Type 2 diabetes

## Abstract

**Background:**

The reno-protective effect of second-line treatments in type 2 diabetes has been assessed by clinical trials but generalizability to routine clinical practice is still uncertain. We aimed to assess the effectiveness of these treatments, when added to metformin, on the risk of chronic kidney disease (CKD).

**Methods:**

A real-world, hospital-based, type 2 diabetes cohort was retrospectively assembled at Ramathibodi Hospital from 2010 to 2019. Patients who received sulfonylureas (SU), thiazolidinediones (TZD), dipeptidyl peptidase-4 inhibitors (DPP4i), or sodium-glucose cotransporter-2 inhibitors (SGLT2i), as second-line antihyperglycemic treatment were included. Treatment effect models with inverse probability weighting and regression adjustment were used to estimate CKD risk according to treatment.

**Results:**

CKD was identified in 4,132 of the 24,777 patients with type 2 diabetes (16.7%). The CKD incidence (95% CI) was 4.1% (2.2%, 6.9%), 13.5% (12.5%, 14.6%), 14.8% (13.5%, 16.1%), and 18.0% (17.4%, 18.5%) for patients receiving SGLT2i, DPP4i, TZD, and SU treatment, respectively. The average treatment effects (i.e., the difference in CKD risk) for SGLT2i, DPP4i, and TZD compared to SU were − 0.142 (− 0.167, − 0.116), − 0.046 (− 0.059, − 0.034), and − 0.004 (− 0.023, 0.014), respectively, indicating a significant reduction in CKD risk of 14.2% and 4.6% in the SGLT2i and DPP4i groups, respectively, compared to the SU group. Furthermore, SGLT2i significantly reduced CKD risk by 13.7% (10.6%, 16.8%) and 9.5% (6.8%, 12.2%) when compared to TZD and DPP4i, respectively.

**Conclusions:**

Our study identified 14.2%, 13.7%, and 9.5% reduced CKD risk in Thai patients with type 2 diabetes who were treated with SGLT2i compared to those treated with SU, TZD, and DPP4i, respectively, in real-world clinical data. Previous evidence of a reno-protective effect of SGLT2i reported in other populations is consistent with our observations in this Southeast Asian cohort.

**Supplementary Information:**

The online version contains supplementary material available at 10.1186/s12933-022-01641-2.

## Background

Type 2 diabetes is increasingly prevalent in terms of global chronic disease burden. The prevalence of type 2 diabetes was estimated at 6,059 cases per 100,000 in 2017, and is expected to reach 7,079 cases per 100,000 by 2030 [1]. Patients with type 2 diabetes have a high risk of developing complications such as retinopathy, nephropathy, neuropathy, and cardiovascular disease (CVD). Management of type 2 diabetes includes lifestyle modifications and pharmacological interventions, which aim to normalize blood glucose levels. The American Diabetes Association (ADA) recommends metformin monotherapy as first-line treatment if there is no contraindication [2]. A second-line antihyperglycemic drug is added to metformin for patients who continue to have uncontrolled glycemic levels or who have any complications such as CVD or chronic kidney disease (CKD). Currently, second-line antihyperglycemic treatment options include sulfonylureas (SU), thiazolidinediones (TZD), dipeptidyl peptidase-4 inhibitors (DPP4i), sodium-glucose cotransporter-2 inhibitors (SGLT2i), glucagon-like peptide-1 receptor agonists (GLP-1 RA), and basal insulin. The selection of a second-line drug is based on many factors including comorbidities, drug efficacy, side effects, costs, patients’ accessibility, and patient-doctor’s preferences.

CKD and CVD represent significant diabetes-related complications [3, 4]. Two more recent drug classes (i.e., SGLT2i and GLP-1 RA) have indicated cardio- and reno-protection in patients with type 2 diabetes. ADA guidelines have recommended SGLT2i or GLP-1 RA, combined with metformin, in patients with type 2 diabetes who have CKD with or without established CVD, or heart failure [2]. Meta-analyses of randomized-controlled trials (RCT) have also supported reno-protective effects of SGLT2i [5–8] and GLP-1 RA [8–10]. In addition, network meta-analyses of RCTs reported that SGLT2i provided a more pronounced reno-protective effect than GLP1-RA [11, 12]. However, these data were generated within the constraints of the ideal conditions of RCTs, e.g., close follow-up and monitoring [8, 13]. These RCTs were also mainly conducted in Caucasian patients who mostly had established CVD and poor baseline kidney function, therefore, the generalizability of the findings from these RCTs in a real-world setting, and for Asian patients in particular, remains uncertain. Therefore, we aimed to investigate the effectiveness of SGLT2i and other second-line antihyperglycemic drugs when added to metformin on the risk of CKD development in a real world, hospital-based cohort of Thai patients with type 2 diabetes.

## Methods

**Fig. 1:**
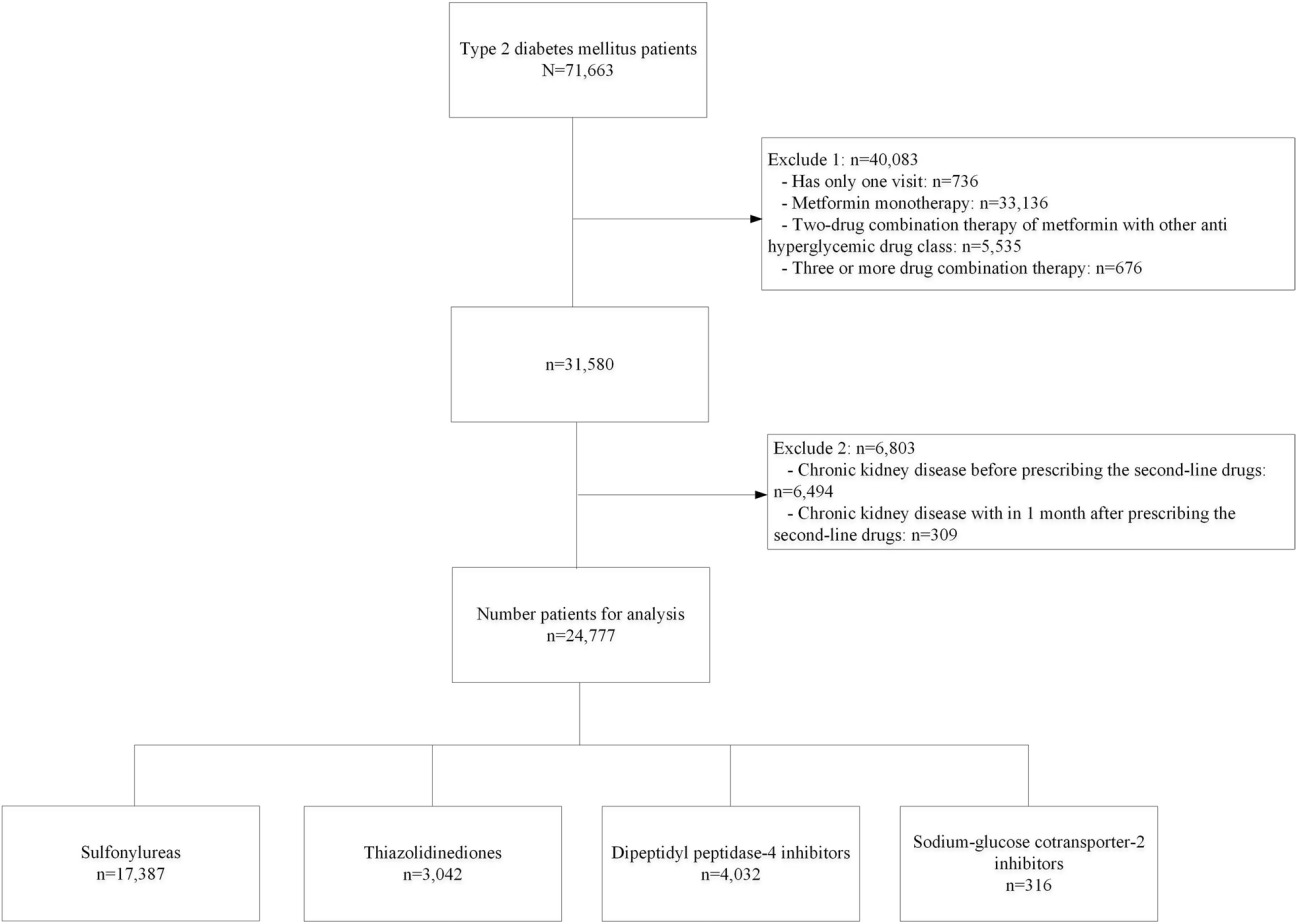
Flow of data for analysis

This study included a retrospective cohort of patients with type 2 diabetes from Ramathibodi Hospital, Bangkok, Thailand, from January 2010 to December 2019. Patients with type 2 diabetes were identified by the International Statistical Classification of Diseases, Ninth and Tenth Revisions (ICD-9 and ICD-10) as shown in Fig. 1. Patients with type 2 diabetes were eligible if they were 18 years or older, and received any of the following second-line antihyperglycemic drugs of interest: SU, TZD, DPP4i, and SGLT2i. Patients were excluded if they received triple combination therapy, or developed CKD prior to, or within one month following receipt of a second-line treatment. The study was approved by the Institutional Review Board of the Faculty of Medicine, Ramathibodi Hospital, Mahidol University (COA. MURA2021/522).

### Treatments, outcomes, and covariables

The second-line antihyperglycemic drugs of interest were SU, TZD, DPP4i, SGLT2i, and GLP1-RA, and were identified from the hospital medication databases. In order to emulate a target trial [14], the index date was defined as the date of initially receiving the second-line drug, and the end date on first report of CKD occurrence, or the date of the last visit if CKD-free. In addition, three treatment approaches were considered as follows: First, an intention-to-treat (ITT) approach considered patients who received the initial second-line treatment during the study period (January 2010–2019), and who were followed to an endpoint (i.e., CKD occurrence or the end of study, whichever occurred first) regardless of switching/changing treatments, i.e. patients who switched second-line drugs during follow-up were analysed as if they still received the initial second-line drug. Second, a per-protocol analysis (PPA) included only patients that consistently received initial second-line treatment throughout the follow-up period, which was evaluated based on their prescriptions and dispensing at each follow-up visit. Third, a modified ITT approach censored patients on the date of the second-line treatment change if they developed CKD after switching treatment.

Demographic data (i.e., age and gender) and body mass index (BMI) were retrieved from electronic medical records. Duration of first-line treatment was defined as the time since first receiving metformin until the index date, i.e., initiation of the second-line treatment. Laboratory parameters (i.e., fasting plasma glucose (FPG), hemoglobin A1c (HbA1c), low-density lipoprotein cholesterol (LDL-C), high-density lipoprotein cholesterol (HDL-C), triglycerides, serum creatinine (SCr), albumin-to-creatinine ratio (ACR), urine dipstick test), and comorbidities (i.e., hypertension (HT), CVD, dyslipidemia (DLP), diabetic retinopathy (DR), and peripheral artery disease (PAD) identified by ICD-9 and ICD-10) were retrieved from laboratory and clinical databases for every follow-up visit. Estimated glomerular filtration rate (eGFR) was calculated using the 2009 CKD-EPI creatinine equation. These baseline characteristics were obtained on the date that the second-line drug was prescribed. However, availability of laboratory data during follow up was inconsistent due to variation in follow-up appointment times (e.g., every 3, 6, or 12 months); these data were therefore grouped into 6-month intervals.

The outcome of interest was CKD, which was identified as follows: ICD-10 (Code N18.1 to N18.9, N083, E112), ICD-9 (Code 3895, 3927, 3942, 3943, 3995, 5498), and/or any of the following laboratory measures on at least three consecutive occasions: eGFR < 60 ml/min/1.73 m^2^, ACR ≥ 30 mg/g, urine dipstick trace or + 1 or above [15].

### Statistical analysis

Missing data ranged from 40 to 62%; multiple imputation by chained equations (MICE) with 40-replications were performed to impute missing data for the whole follow-up time on the assumption that data were missing at random, see Additional file 1: Table S1. Baseline characteristics were described by treatment group as a number (percentage) for categorical variables and mean (SD) or median (range) for continuous variables. Variables were compared by treatment groups using Chi-squared or analysis of variance tests respectively.

Treatment effect models with inverse probability weighting and regression adjustment (IPWRA) were used to estimate the effects of each second-line drug on the development of CKD. Laboratory measures and underlying diseases identified at initiation of the second line drug (the index date) were used in IPWRA. The analyses were performed as per the following steps:

- First, the treatment model (TM) was constructed using multinomial logistic regression by regressing treatments on variables that may have been associated with treatment allocation including age, sex, BMI, FPG, HbA1c, eGFR, HT, CVD, DLP, LDL-C, HDL-C, triglycerides, and statin medication. A likelihood ratio test was used to identify significant variables which were retained in the final TM and a propensity score was estimated accordingly.
- Second, the outcome model (OM) was constructed using a logit equation by weighting by inverse probability of treatment allocation (i.e., a propensity score) estimated from the TM. Confounders considered in the TM were also included, but only significant variables were retained in the OM. Finally, potential outcome mean (POM) (i.e., the risks of CKD development associated with each second-line treatment group) was estimated. Average treatment effect (ATE) (i.e., the difference between two POMs), and 95% confidence intervals (CI) were estimated. All possible comparisons between second-line drug classes of interest were performed.

Treatment effect models require several assumptions [16]. First, the conditional mean independence assumption was tested, i.e., if variables associated with treatment allocation were balanced between treatment groups, i.e., their weighted standardized mean differences were less than 0.2 and the variance ratios were close to 1. Second, an overlap assumption was verified, i.e., if the probability of receiving treatments overlapped between groups.

All analyses were performed using STATA version 17.0. (Stata Corp., College Station, TX, USA). A P-value < 0.05 was considered statistically significant.

## Results

### Baseline characteristics

Of 71,663 patients identified, 40,083 were initially excluded for the following reasons: patients had only a single visit, received metformin monotherapy, received metformin plus other antihyperglycemic drugs not included in our list of interest (e.g., alpha glucosidase inhibitors or insulin), or received triple combination therapy. Of 31,580 remaining patients, 6,803 were excluded due to the occurrence of CKD before or within one month following receipt of the second-line medication, leaving 24,777 patients for analysis with mean (SD) follow-up time of 4.1 (3.1) years, see Fig. 1. There were 17,387 (70.2%), 3,042 (12.3%), 4,032 (16.3%), 316 (1.3%), and 185 (0.7%) patients who received either SU, TZD, DPP4i, SGLT2i, and GLP1-RA, respectively. Finally, those in receipt of GLP1-RA were not included in the analysis due to the small number of patients and outcomes available.

Most baseline patient characteristics differed across treatment groups, see Table 1. DPP4i patients tended to be older, were more likely to be female and hypertensive; the SU group had higher mean FPG and HbA1c, lower mean eGFR, higher mean ACR, and a higher proportion of DLP. Those in receipt of SGLT2i tended to have a higher mean BMI and eGFR, lower mean ACR, and were more likely to have a history of CVD and hypertension. In addition, patients in the SGLT2i group had longer duration of first-line treatment than the other groups, except TZD.

**Table 1:** Baseline characteristics of second-line antihyperglycemic drugs

Characteristics	SU	TZD	DPP4i	SGLT2i	P-value
Number of patients, n (%)	17,387 (70.2)	3,042 (12.3)	4,032 (16.3)	316 (1.3)	
Age, year, mean (SD)	61.2 (11.9)	59.7 (11.7)	64.1 (11.9)	59.1 (12.6)	< 0.001
*Sex, n (%)*					
Female	10,053 (57.8)	1,752 (57.6)	2,408 (59.7)	169 (53.5)	0.046
Male	7,334 (42.2)	1,290 (42.4)	1,624 (40.3)	147 (46.5)	
BMI, kg/m^2^, mean (SD)	27.2 (4.5)	28.5 (5.2)	27.3 (4.7)	30.2 (5.9)	< 0.001
FPG, mg/dL, mean (SD)	180.0 (77.1)	165.1 (69.6)	170.2 (73.2)	154.5 (48.6)	< 0.001
HbA1c, %, mean (SD)	8.2 (1.8)	8.0 (1.7)	8.0 (1.7)	7.7 (1.5)	< 0.001
eGFR, ml/min/1.73 m^2^, mean (SD)	75.8 (24.6)	78.4 (25.0)	76.7 (23.8)	87.0 (20.5)	< 0.001
ACR, mg/g, median (IQR)	18.4 (7.0, 71.0)	12.7 (6.0, 42.1)	12.7 (5.8, 39.5)	9.8 (4.8, 40.9)	< 0.001
*HT, n (%)*					
Yes	13,958 (80.3)	2,444 (80.3)	3,428 (85.0)	269 (85.1)	< 0.001
No	3,429 (19.7)	598 (19.7)	604 (15.0)	47 (14.9)	
*ACEi/ARB, n (%)*					
Yes	8,408 (48.4)	1,528 (50.2)	1,988 (49.3)	167 (52.8)	0.096
No	8,979 (51.6)	1,514 (49.8)	2,044 (50.7)	149 (47.2)	
*CVD, n (%)*					
Yes	1,319 (7.6)	149 (4.9)	567 (14.1)	87 (27.5)	< 0.001
No	16,068 (92.4)	2,893 (95.1)	3,465 (85.9)	229 (72.5)	
*PAD, n (%)*					
Yes	190 (1.1)	41 (1.3)	68 (1.7)	5 (1.6)	0.016
No	17,197 (98.9)	3,001 (98.7)	3,964 (98.3)	311 (98.4)	
*DR, n (%)*					
Yes	476 (2.7)	108 (3.6)	119 (3.0)	9 (2.8)	0.100
No	16,911 (97.3)	2,934 (96.4)	3,913 (97.0)	307 (97.2)	
*DLP, n (%)*					
Yes	11,738 (67.5)	1,919 (63.1)	2,305 (57.2)	162 (51.3)	< 0.001
No	5,649 (32.5)	1,123 (36.9)	1,727 (42.8)	154 (48.7)	
*Statin, n (%)*					
Yes	12,049 (69.3)	2,270 (74.6)	3,023 (75.0)	245 (77.5)	< 0.001
No	5,338 (30.7)	772 (25.4)	1,009 (25.0)	71 (22.5)	
LDL-C, mg/dL, mean (SD)	117.8 (36.0)	115.2 (34.5)	111.6 (37.0)	107.6 (37.9)	< 0.001
Triglycerides, mg/dL, median (IQR)	147.1 (118.0, 189.0)	138.9 (111.0, 177.0)	142.0 (109.9, 186.1)	140.0 (108.0, 188.8)	< 0.001
HDL-C, mg/dL, mean (SD)	45.8 (10.7)	46.7 (10.9)	45.8 (11.3)	44.0 (10.2)	< 0.001
Duration of first-line treatment, month, median (IQR)	4.59 (0.03, 46.82)	19.31 (0.03, 57.74)	7.02 (0.03, 48.16)	13.72 (0.03, 54.36)	< 0.001

ACEi: Angiotensin-converting enzyme inhibitors; ACR: Albumin-to-creatinine ratio; ARB: Angiotensin receptor blockers; BMI: Body mass index; CVD: Cardiovascular disease; DLP: Dyslipidemia; DPP4i: Dipeptidyl peptidase-4 inhibitors; DR: Diabetic retinopathy; eGFR: Estimated glomerular filtration rate; FPG: Fasting plasma glucose; HbA1c: Hemoglobin A1c; HDL-C: High density lipoprotein cholesterol; HT: Hypertension; IQR: Interquartile range; LDL-C: Low density lipoprotein cholesterol; PAD: Peripheral artery disease; SD: Standard deviation; SGLT2i: Sodium-glucose cotransporter-2 inhibitors; SU: Sulfonylureas; TZD: Thiazolidinediones

### Treatment model

Variables that were significantly associated with second-line drug allocation included age, sex, BMI, FPG, eGFR, HT, CVD, LDL-C, HDL-C, triglycerides, and statin use, see Additional file 1: Table S2. These variables including use of angiotensin-converting enzyme inhibitors (ACEi) or angiotensin receptor blockers (ARB) were retained in the TM to estimate the propensity score. Before weighting by inverse propensity score, the absolute standardized mean differences ranged from 0.0012 to 0.2837, 0.0031 to 0.2436, and 0.0259 to 0.5720 for TZD vs. SU, DPP4i vs. SU, and SGLT2i vs. SU, respectively (see Table 2). After weighting by inverse propensity score, the absolute standardized mean differences were close to zero, i.e., ranging from 0.0004 to 0.0803, 0.0003 to 0.0227, and 0.0026 to 0.1604; variance ratios were, in the main, close to one (see balance plots, Additional file 1: Figure S1), confirming that these variables were comparable across treatment groups and overlapping with positive probabilities (see Additional file 1: Figure S2).

**Table 2:** Estimated standardized mean difference between treatment groups before and after propensity score weighting

Variables	Standardized differences		Variance ratio	
	Raw	Weighted	Raw	Weighted
*TZD vs. SU*				
Age, year	− 0.1263	− 0.0201	0.9809	1.0076
Male vs. Female	0.0043	− 0.0027	1.0016	0.9991
BMI, kg/m^2^	0.2837	− 0.0231	1.3183	0.9799
ln(FPG), mg/dL	− 0.2419	0.0511	0.8574	1.2458
HT	0.0012	− 0.0129	0.9984	1.0205
eGFR group < 90 ml/min/1.73 m^2^	− 0.1037	− 0.0084	1.0659	1.0057
CVD	− 0.1110	− 0.0004	0.6654	0.9989
Statin	0.1172	− 0.0205	0.8915	1.0186
HDL-C, mg/dL	0.0847	− 0.0100	1.0447	1.0442
Triglycerides, mg/dL	− 0.0717	0.0803	1.0053	8.9630
LDL-C, mg/dL	− 0.0729	− 0.0042	0.9187	1.0439
ACEi/ARB	0.0373	− 0.0142	1.0013	0.9991
*DPP4i vs. SU*				
Age, year	0.2436	− 0.0111	1.0056	1.0908
Male vs. Female	− 0.0374	0.0074	0.9870	1.0023
BMI, kg/m^2^	0.0280	0.0019	1.0991	1.0250
ln(FPG), mg/dL	− 0.1617	0.0065	0.9541	1.1660
HT	0.1247	0.0027	0.8056	0.9957
eGFR group < 90 ml/min/1.73 m^2^	− 0.0207	0.0003	1.0149	0.9998
CVD	0.2104	− 0.0020	1.7271	0.9940
Statin	0.1275	− 0.0080	0.8810	1.0073
HDL-C, mg/dL	− 0.0031	− 0.0053	1.1262	1.1293
Triglycerides, mg/dL	− 0.0624	0.0109	0.7698	1.1319
LDL-C, mg/dL	− 0.1704	0.0227	1.0567	1.2859
ACEi/ARB	0.0200	0.0015	1.0011	1.0001
*SGLT2i vs. SU*				
Age, year	− 0.1716	0.0036	1.1340	0.9964
Male vs. Female	0.0872	0.0154	1.0233	1.0048
BMI, kg/m^2^	0.5720	0.0204	1.6763	1.1053
ln(FPG), mg/dL	− 0.4200	0.1604	0.6622	1.1047
HT	0.1278	0.0657	0.8029	0.8939
eGFR group < 90 ml/min/1.73 m^2^	− 0.3782	− 0.0722	1.1352	1.0444
CVD	0.5425	0.0225	2.8544	1.0672
Statin	0.1855	0.1306	0.8224	0.8670
HDL-C, mg/dL	− 0.1733	0.0041	0.9205	1.1982
Triglycerides, mg/dL	− 0.0259	− 0.1194	1.0624	0.5776
LDL-C, mg/dL	− 0.2757	− 0.0246	1.1049	1.3047
ACEi/ARB	0.0897	0.0026	1.0009	1.0001

ACEi: Angiotensin-converting enzyme inhibitors; ARB: Angiotensin receptor blockers; BMI: Body mass index; CVD: Cardiovascular disease; DPP4i: Dipeptidyl peptidase-4 inhibitors; eGFR: Estimated glomerular filtration rate; FPG: Fasting plasma glucose; HDL-C: High density lipoprotein cholesterol; HT: Hypertension; LDL-C: Low density lipoprotein cholesterol; SGLT2i: Sodium-glucose cotransporter-2 inhibitors; SU: Sulfonylureas; TZD: Thiazolidinediones

### CKD events and outcome model

CKD was identified in 4,132 of the 24,777 patients (16.7%). The incidence (95% CI) of CKD by treatment group was 4.1% (2.2%, 6.9%), 13.5% (12.5%, 14.6%), 14.8% (13.5%, 16.1%), and 18.0% (17.4%, 18.5%) for SGLT2i, DPP4i, TZD, and SU, respectively.

Variables significantly associated with CKD and thus remaining in the OM were age, sex, duration of first-line treatment, HT, PAD, DR, and HDL-C, see Additional file 1: Table S2. Results for all IPWRA approaches are described in Tables 3 and 4. For the ITT approach, the POMs (95% CI), i.e., the risk of CKD development in SGLT2i, DPP4i, TZD, and SU groups were 0.037 (0.012, 0.063), 0.133 (0.122, 0.143), 0.175 (0.157, 0.193), and 0.179 (0.173, 0.185), respectively, see Table 3. The ATE (i.e., the difference of POM) of SGLT2i, DPP4i, and TZD compared to SU were − 0.142 (− 0.167, − 0.116), − 0.046 (− 0.059, − 0.034), and − 0.004 (− 0.023, 0.014), respectively, representing significant reductions in CKD risk of 14.2% and 4.6% in the SGLT2i and DPP4i groups, respectively, compared to the SU group, with no significant difference between TZD and SU groups, see Table 4. In addition, CKD risk was 13.7% (10.6%, 16.8%) and 4.2% (2.1%, 6.3%) significantly lower in SGLT2i and DPP4i when compared to TZD. Furthermore, SGLT2i significantly reduced CKD risk by 9.5% (6.8%, 12.2%) when compared to DPP4i.

**Table 3:** Estimation of potential outcome means between second − line drugs: Treatment effect model with inverse probability weighting and regression adjustment

Treatment	POM	Lower limit	Upper limit
*ITT*			
SGLT2i	0.037	0.012	0.063
DPP4i	0.133	0.122	0.143
TZD	0.175	0.157	0.193
SU	0.179	0.173	0.185
*PPA*			
SGLT2i	0.024	0.002	0.046
DPP4i	0.146	0.132	0.161
TZD	0.194	0.172	0.216
SU	0.201	0.193	0.208
*Modified ITT*			
SGLT2i	0.037	0.012	0.063
DPP4i	0.133	0.122	0.143
TZD	0.175	0.157	0.193
SU	0.179	0.173	0.185

DPP4i: Dipeptidyl peptidase-4 inhibitors; ITT: Intention-to-reat; POM: Potential outcome mean; PPA: Per-protocol analysis; SGLT2i: Sodium-glucose cotransporter-2 inhibitors; SU: Sulfonylureas; TZD: Thiazolidinediones

**Table 4:** Estimation of treatment effects between second − line drugs: Treatment effect model with inverse probability weighting and regression adjustment

	ATE (95% CI)				
	Treatment	SU	TZD	DPP4i	SGLT2i
**RR (95% CI)**	*ITT*				
	SU	Ref.	− 0.004 (− 0.023, 0.014)	− 0.046 (− 0.059, − 0.034)	− 0.142 (− 0.167, − 0.116)
	TZD	0.98 (0.87, 1.08)	Ref.	− 0.042 (− 0.063, − 0.021)	− 0.137 (− 0.168, − 0.106)
	DPP4i	0.74 (0.68, 0.81)	0.76 (0.66, 0.86)	Ref.	− 0.095 (− 0.122, − 0.068)
	SGLT2i	0.21 (0.07, 0.35)	0.21 (0.07, 0.36)	0.28 (0.09, 0.47)	Ref.
	*PPA*				
	SU	Ref.	− 0.007 (− 0.030, 0.016)	− 0.055 (− 0.071, − 0.039)	− 0.177 (− 0.200, − 0.154)
	TZD	0.96 (0.85, 1.08)	Ref.	− 0.074 (− 0.021, − 0.055)	− 0.201 (− 0.139, − 0.055)
	DPP4i	0.73 (0.65, 0.80)	0.75 (0.64, 0.87)	Ref.	− 0.149 (− 0.096, − 0.055)
	SGLT2i	0.12 (0.01, 0.23)	0.12 (0.01, 0.24)	0.16 (0.01, 0.32)	Ref.
	*Modified − ITT*				
	SU	Ref.	− 0.004 (− 0.023, 0.014)	− 0.046 (− 0.059, − 0.034)	− 0.142 (− 0.167, − 0.116)
	TZD	0.98 (0.87, 1.08)	Ref.	− 0.042 (− 0.063, − 0.021)	− 0.137 (− 0.168, − 0.106)
	DPP4i	0.74 (0.68, 0.81)	0.76 (0.66, 0.86)	Ref.	− 0.095 (− 0.122, − 0.068)
	SGLT2i	0.21 (0.07, 0.35)	0.21 (0.07, 0.36)	0.28 (0.09, 0.47)	Ref.

ATE: Average treatment effect; CI: Confidence interval; DPP4i: Dipeptidyl peptidase-4 inhibitors; ITT: Intention-to-treat; PPA: Per-protocol analysis; Ref.: Reference; RR: Relative risk; SGLT2i: Sodium-glucose cotransporter-2 inhibitors; SU: Sulfonylureas; TZD: Thiazolidinediones

The PPA approach showed similar trends in treatment effects as ITT with POMs in SGLT2i, DPP4i, TZD, and SU groups of 0.024 (0.002, 0.046) 0.146 (0.132, 0.161), 0.194 (0.172, 0.216), and 0.201 (0.193, 0.208), respectively. The ATEs of SGLT2i, DPP4i, and TZD compared to SU were − 0.177 (− 0.200, − 0.154), − 0.055 (− 0.071, − 0.039), and − 0.007 (− 0.030, 0.016), respectively, representing significant reductions in CKD risk of 17.7% and 5.5% in the SGLT2i and DPP4i compared to the SU groups. In addition, SGLT2i significantly reduced CKD risk by 20.1% and 14.9% compared to TZD and DPP4i, respectively. The modified ITT yielded similar results as the ITT approach.

## Discussion

We emulated a target trial setting using real-world data from a cohort of Thai patients with type 2 diabetes to estimate treatment effectiveness of second-line antihyperglycemic drugs on CKD progression. Three analytic approaches with IPWRA (i.e., ITT, PPA, and modified ITT) were applied to evaluate treatment patterns that were subject to significant variation among recognized CKD risk factors. Our findings indicated 14.2%, 13.7%, and 9.5% lower CKD risk associated with SGLT2i compared to SU, TZD, and DPP4i, respectively.

We compared treatment effectiveness of SGLT2i on renal outcomes with other second-line drugs by utilizing real-world data from Ramatibodi Hospital, Bangkok, Thailand. Although SGLT2i have been available since 2015, treatment care and patient characteristics before and after 2015 would be expected to be similar with minimal impact on the outcomes assessed. A sensitivity analysis performed on data from 2015 showed similar trends of SGLT2i effect, although the treatment effect of SGLT2i vs. SU was reduced by about a half, see Additional file 1: Table S3.

Our findings support the SGLT2i reno-protective effects reported in previous RCTs [5–8] and real-world studies [17–20]. A recent meta-analysis that included five major cardiovascular outcome trials revealed that SGLT2i (i.e., empagliflozin, canagliflozin, dapagliflozin, and ertugliflozin) were associated with a lower risk of composite kidney outcomes (i.e., doubling of serum creatinine level, a 40% reduction in eGFR, end-stage renal disease, renal-replacement therapy, and renal death) with an overall pooled hazard ratio [HR (95% CI)] of 0.62 (0.56, 0.70) [6]. For real-world evidence, a large multinational cohort study (CVD-REAL 3) with propensity score matching observed that patients treated with SGLT2i had a significant lower risk of kidney function decline approximating 50%, compared to patients treated with other antihyperglycemic drugs [17]. A propensity score-matching cohort study from Scandinavian countries also showed an association between SGLT2i and lower risk of composite kidney outcomes (i.e., renal-replacement therapy, renal death, and hospitalization for renal events) than DPP4i with a HR of 0.42 (0.34, 0.53) [18]. Another propensity score-matching cohort study undertaken across three Taiwanese electronic health record databases also confirmed that SGLT2i treatment significantly lowered eGFR risk by 30%, 40%, and 50% in patients with type 2 diabetes across different baseline eGFR levels [19]. Of note, our findings were similar to a recent study by Xie and colleagues [20] that compared the effectiveness of SGLT2i, GLP-1 RA, DPP4i and SU on composite renal outcomes (eGFR decline by more than 50%, end-stage kidney disease, and all-cause mortality) in United States veterans, showing significant SGLT2i reno-protective effects compared to SU and DPP4i with a HR of 0.68 (0.63, 0.74) and 0.76 (0.70, 0.82), respectively. Their study also reported a significant DPP4i protective effect with a HR of 0.90 (0.86, 0.95) compared to SU, and no significant difference between SGLT2i and GLP-1 RA. In contrast, we were unable to evaluate the effects of GLP1-RA given insufficient data for GLP1-RA was available in our cohort, although of note, their study was significantly larger.

Multiple biological mechanisms have been proposed to contribute to the reno-protective effects associated with SGLT2i, especially the restoration of tubuloglomerular feedback [21]. SGLT2i reduce reabsorption of glucose and sodium at the proximal tubule by inhibiting sodium-glucose co-transporter-2 leading to glucosuria and natriuresis, and also increased sodium delivery to the macula densa. This has been shown to induce adenosine release causing afferent arteriole constriction and subsequent reduction of intraglomerular pressure and glomerular hyperfiltration [21]. Inhibition of sodium-glucose co-transporter-2 also reduces the tubular workload and hypoxia which could lead to CKD [22]. Furthermore, SGLT2i decrease renal inflammation by lowering inflammatory cytokines and chemokines such as interleukin-6 (IL-6), tumor necrosis factor-alpha (TNF-α), and monocyte chemoattractant protein-1 (MCP-1) [23–25]. These mechanisms likely contribute to the overall reno-protective effects associated with SGLT2i.

Our study had several strengths. The treatment effect models with IPWRA were suitable to address the imbalance of risk factors associated with second-line antihyperglycemic drug allocation and the development and progression of CKD. The application of inverse propensity weighting helped redress risk factor imbalance making comparisons of real-world data more in line with an RCT. This large hospital derived cohort reflects real clinical practice and as such better generalizability to real-world settings. The 10-year follow-up time is considered sufficiently long to observe CKD progression. We also performed ITT, PPA, and modified ITT approaches to emulate a target trial and the reno-protective effect associated with SGLT2i was consistent across all three approaches. To our knowledge, this is one of only several studies to confirm a reno-protective effect in a Southeast Asian population. However, our study had some limitations. GLP1-RA treatment was not included in the analysis due to insufficient data. Studies that include larger numbers of participants treated with GLP1-RA should be undertaken to evaluate its effect on reno-protective outcomes. In addition, despite the nature of our approach to adjust for potential confounding, some residual confounding may remain due to the observational nature of our study and the fact that other factors that might have influenced treatment decisions, such as socio-economic constraints, private health insurance etc., were unavailable. Although we did not apply a competing risk analysis by considering all-cause mortality as a competing risk event, a sensitivity analysis that excluded patients who died before CKD provided similar findings to the primary analysis, see Additional file 1: Table S4.

## Conclusions

In conclusion, our study identified 14.2%, 13.7%, and 9.5% lower CKD risk in Thai patients with type 2 diabetes associated with SGLT2i treatment use compared to SU, TZD, and DPP4i, respectively, from real-world clinical data. Previous evidence of a reno-protective effect of SGLT2i seen in other populations is also consistent in this Southeast Asian cohort.

## Supplementary Information

**Additional file 1.** Supplementary Tables S1–S4 and Supplementary Figures S1–S2.

## Data Availability

All data generated or analysed during this study are included in this published article and its supplementary information files. The datasets used and/or analysed during the current study are available from the corresponding author on reasonable request.

## Abbreviations

ACEi: Angiotensin-converting enzyme inhibitors
ACR: Albumin-to-creatinine ratio
ADA: The American Diabetes Association
ARB: Angiotensin receptor blockers
ATE: Average treatment effect
BMI: Body mass index
CI: Confidence interval
CKD: Chronic kidney disease
CVD: Cardiovascular disease
DLP: Dyslipidemia
DPP4i: Dipeptidyl peptidase-4 inhibitors
DR: Diabetic retinopathy
eGFR: Estimated glomerular filtration rate
FPG: Fasting plasma glucose
GLP1-RA: Glucagon-like peptide-1 receptor agonists
HDL-C: High-density lipoprotein cholesterol
HR: Hazard ratio
HT: Hypertension
HbA1c: Hemoglobin A1c
ICD: The International Statistical Classification of Diseases
IL-6: Interleukin-6
IPWRA: Inverse probability weighting and regression adjustment
IQR: Interquartile range
ITT: Intention-to-treat
LDL-C: Low-density lipoprotein cholesterol
MCP-1: Monocyte chemoattractant protein-1
MICE: Multiple Imputation by Chained Equations
OM: Outcome model
PAD: Peripheral artery disease
POM: Potential outcome mean
PPA: Per-protocol analysis
RCT: Randomized-controlled trials
SCr: Serum creatinine
SD: Standard deviation
SGLT2i: Sodium-glucose cotransporter-2 inhibitors
SU: Sulfonylureas
TM: Treatment model
TNF-α: Tumor necrosis factor-alpha
TZD: Thiazolidinediones
