# Supplementary material for: Effects of second-line antihyperglycemic drugs on the risk of chronic kidney disease: applying a target trial approach to a hospital-based cohort of Thai patients with type 2 diabetes
Source: Cardiovasc Diabetol. 2022 Nov 17;21:248. doi: 10.1186/s12933-022-01641-2 (PMC9670521; doi:10.1186/s12933-022-01641-2)
Supplement: Supplementary file 1 — Additional file 1. Supplementary Tables S1–S4 and Supplementary Figures S1–S2. [file 12933_2022_1641_MOESM1_ESM.docx]

Table S1. Numbers and percentages of missing data

| Variables | Total n | Missing data, n (%) | |
| --- | --- | --- | --- |
|  |  | Before imputation | After imputation |
| Age, year | 24,777 | 0 (0.00) | 0 (0.00) |
| Sex | 24,777 | 0 (0.00) | 0 (0.00) |
| BMI, kg/m^2^ | 24,777 | 15,355 (61.98) | 24 (0.10) |
| FPG, mg/dL | 24,777 | 9,904 (39.97) | 14 (0.06) |
| HbA1c, % | 24,777 | 11,352 (45.82) | 29 (0.12) |
| eGFR, ml/min/1.73 m^2^ | 24,777 | 10,293 (41.54) | 9 (0.04) |
| HT | 24,777 | 0 (0.00) | 0 (0.00) |
| CVD | 24,777 | 0 (0.00) | 0 (0.00) |
| PAD | 24,777 | 0 (0.00) | 0 (0.00) |
| DR | 24,777 | 0 (0.00) | 0 (0.00) |
| DLP | 24,777 | 0 (0.00) | 0 (0.00) |
| LDL-C, mg/dL | 24,777 | 12,219 (49.32) | 41 (0.17) |
| HDL-C, mg/dL | 24,777 | 13,671 (55.18) | 44 (0.18) |
| Triglycerides, mg/dL | 24,777 | 12,324 (49.74) | 33 (0.13) |
| ACEi/ARB | 24,777 | 0 (0.00) | 0 (0.00) |
| Statin | 24,777 | 0 (0.00) | 0 (0.00) |

^ACEi: Angiotensin-converting enzyme inhibitors; ARB: Angiotensin receptor blockers; BMI: body mass index; CVD: Cardiovascular disease; DLP: Dyslipidemia; DR: Diabetic retinopathy; eGFR: Estimated glomerular filtration rate; FPG: Fasting plasma glucose; HbA1c: Hemoglobin A1c; HDL-C: High density lipoprotein cholesterol; HT: Hypertension; LDL-C: Low density lipoprotein cholesterol; PAD: Peripheral artery disease^

Table S2. Variables retained in the treatment model and outcome model

| Treatment model | | | Outcome model | | |
| --- | --- | --- | --- | --- | --- |
| Variables | OR (95% CI) | P-value | Variables | HR (95% CI) | P-value |
| SGLT2i vs. SU |  |  | Second-line drug |  |  |
| Age, year | 1.006 (0.994, 1.018) | 0.344 | SGLT2i | 0.569 (0.330, 0.981) | 0.043 |
| Male vs. Female | 1.129 (0.887, 1.438) | 0.325 | DPP4i | 0.847 (0.773, 0.928) | <0.001 |
| BMI, kg/m^2^ | 1.112 (1.090, 1.134) | <0.001 | TZD | 0.878 (0.794, 0.969) | 0.010 |
| ln(FPG), mg/dL | 0.206 (0.134, 0.315) | <0.001 | SU | 1 |  |
| HT | 1.009 (0.700, 1.454) | 0.961 | Age, year | 1.037 (1.034, 1.040) | <0.001 |
| eGFR < 90 ml/min/1.73 m^2^ | 0.349 (0.268, 0.454) | <0.001 | Male vs. Female | 1.295 (1.215, 1.381) | <0.001 |
| CVD | 5.062 (3.848, 6.660) | <0.001 | HT | 1.607 (1.453, 1.777) | <0.001 |
| Statin | 1.263 (0.955, 1.671) | 0.102 | HDL-C, mg/dL | 0.982 (0.978, 0.985) | <0.001 |
| HDL-C, mg/dL | 0.992 (0.980, 1.004) | 0.204 | PAD | 1.506 (1.165, 1.946) | 0.002 |
| Triglycerides, mg/dL | 1.0001 (0.9993, 1.0008) | 0.870 | DR | 1.902 (1.628, 2.221) | <0.001 |
| LDL-C, mg/dL | 0.993 (0.990, 0.997) | <0.001 | Duration of first-line treatment, month | 0.997 (0.996, 0.998) | <0.001 |
| ACEi/ARBs | 0.923 (0.715, 1.193) | 0.542 |  |  |  |
| DPP4ivs. SU |  |  |  |  |  |
| Age, year | 1.027 (1.023, 1.031) | <0.001 |  |  |  |
| Male vs. Female | 0.976 (0.905, 1.051) | 0.520 |  |  |  |
| BMI, kg/m^2^ | 1.025 (1.017, 1.033) | <0.001 |  |  |  |
| ln(FPG), mg/dL | 0.681 (0.606, 0.766) | <0.001 |  |  |  |
| HT | 1.146 (1.027, 1.279) | 0.015 |  |  |  |
| eGFR < 90 ml/min/1.73 m^2^ | 0.631 (0.579, 0.687) | <0.001 |  |  |  |
| CVD | 1.860 (1.667, 2.076) | <0.001 |  |  |  |
| Statin | 1.208 (1.113, 1.310) | <0.001 |  |  |  |
| HDL-C, mg/dL | 0.999 (0.995, 1.002) | 0.432 |  |  |  |
| Triglycerides, mg/dL | 1.0000 (0.9997, 1.0003) | 0.912 |  |  |  |
| LDL-C, mg/dL | 0.997 (0.996, 0.998) | <0.001 |  |  |  |
| ACEi/ARBs | 0.867 (0.802, 0.938) | <0.001 |  |  |  |
| TZD vs. SU |  |  |  |  |  |
| Age, year | 0.995 (0.991, 0.999) | 0.023 |  |  |  |
| Male vs. Female | 1.138 (1.047, 1.237) | 0.002 |  |  |  |
| BMI, kg/m^2^ | 1.059 (1.050, 1.067) | <0.001 |  |  |  |
| ln(FPG), mg/dL | 0.451 (0.393, 0.519) | <0.001 |  |  |  |
| HT | 0.933 (0.830, 1.048) | 0.243 |  |  |  |
| eGFR < 90 ml/min/1.73 m^2^ | 0.816 (0.744, 0.896) | <0.001 |  |  |  |
| CVD | 0.667 (0.558, 0.797) | <0.001 |  |  |  |
| Statin | 1.290 (1.178, 1.413) | <0.001 |  |  |  |
| HDL-C, mg/dL | 1.009 (1.005, 1.013) | <0.001 |  |  |  |
| Triglycerides, mg/dL | 0.9997 (0.9993, 1.0001) | 0.090 |  |  |  |
| LDL-C, mg/dL | 0.999 (0.997, 1.000) | 0.023 |  |  |  |
| ACEi/ARBs | 1.010 (0.922, 1.106) | 0.836 |  |  |  |

^ACEi: Angiotensin-converting enzyme inhibitors; ARB: Angiotensin receptor blockers; BMI: Body mass index; CI: Confidence interval; CVD: Cardiovascular disease; DPP4i: Dipeptidyl peptidase-4 inhibitors; DR: Diabetic retinopathy; eGFR: Estimated glomerular filtration rate; FPG: Fasting plasma glucose; HDL-C: High density lipoprotein cholesterol; HR: Hazard ratio; HT: Hypertension; LDL-C: Low density lipoprotein cholesterol; PAD: Peripheral artery disease; OR: Odds ratio: SGLT2i: Sodium-glucose cotransporter-2 inhibitors; SU: Sulfonylureas; TZD: Thiazolidinediones^

Table S3. Estimation of potential outcome means and average treatment effects between second-line drugs: A sensitivity analysis based on data available from 2015

1. Potential outcome means

| Treatment | POM | Lower limit | Upper limit |
| --- | --- | --- | --- |
| ITT |  |  |  |
| SGLT2i | 0.033 | 0.012 | 0.054 |
| DPP4i | 0.100 | 0.085 | 0.112 |
| TZD | 0.092 | 0.072 | 0.113 |
| SU | 0.108 | 0.098 | 0.117 |
| PPA |  |  |  |
| SGLT2i | 0.025 | 0.003 | 0.046 |
| DPP4i | 0.103 | 0.087 | 0.120 |
| TZD | 0.094 | 0.069 | 0.118 |
| SU | 0.118 | 0.106 | 0.129 |
| Modified ITT |  |  |  |
| SGLT2i | 0.033 | 0.012 | 0.054 |
| DPP4i | 0.100 | 0.085 | 0.112 |
| TZD | 0.092 | 0.072 | 0.113 |
| SU | 0.108 | 0.098 | 0.117 |

^DPP4i: Dipeptidyl peptidase-4 inhibitors; ITT: Intention-to-treat; POM: Potential outcome mean; PPA: Per-protocol analysis; SGLT2i: Sodium-glucose cotransporter-2 inhibitors; SU: Sulfonylureas; TZD: Thiazolidinediones^

1. Average treatment effects

| **RR**  **(95% CI)** | **ATE (95% CI)** | | | | |
| --- | --- | --- | --- | --- | --- |
|  | Treatment | SU | TZD | DPP4i | SGLT2i |
|  | ITT |  |  |  |  |
|  | SU | ref | -0.015 (-0.038, 0.008) | -0.008 (-0.025, 0.008) | -0.074 (-0.097, -0.052) |
|  | TZD | 0.86 (0.65, 1.07) | ref | 0.007 (-0.018, 0.031) | -0.059 (-0.089, -0.030) |
|  | DPP4i | 0.92 (0.77, 1.07) | 1.07 (0.79, 1.35) | ref | -0.066 (-0.091, -0.041) |
|  | SGLT2i | 0.31 (0.11, 0.50) | 0.36 (0.12, 0.60) | 0.34 (0.12, 0.55) | ref |
|  | PPA |  |  |  |  |
|  | SU | ref | -0.024 (-0.051, 0.003) | -0.014 (-0.034, 0.006) | -0.093 (-0.117, -0.069) |
|  | TZD | 0.80 (0.57, 1.02) | ref | 0.010 (-0.020, 0.039) | -0.069 (-0.102, -0.036) |
|  | DPP4i | 0.88 (0.72, 1.04) | 1.11 (0.77, 1.45) | ref | -0.079 (-0.106, -0.052) |
|  | SGLT2i | 0.21 (0.03, 0.39) | 0.26 (0.02, 0.50) | 0.24 (0.03, 0.45) | ref |
|  | Modified-ITT |  |  |  |  |
|  | SU | ref | -0.015 (-0.038, 0.008) | -0.008 (-0.025, 0.008) | -0.074 (-0.097, -0.052) |
|  | TZD | 0.86 (0.65, 1.07) | ref | 0.007 (-0.018, 0.031) | -0.059 (-0.089, -0.030) |
|  | DPP4i | 0.92 (0.77, 1.07) | 1.07 (0.79, 1.35) | ref | -0.066 (-0.091, -0.041) |
|  | SGLT2i | 0.31 (0.11, 0.50) | 0.36 (0.12, 0.60) | 0.34 (0.12, 0.55) | ref |

^ATE: Average treatment effect; CI: Confidence interval; DPP4i: Dipeptidyl peptidase-4 inhibitors; ITT: Intention-to-treat; PPA: Per-protocol analysis; ref: Reference; RR: Relative risk; SGLT2i: Sodium-glucose cotransporter-2 inhibitors; SU: Sulfonylureas; TZD: Thiazolidinediones^

Table S4. Estimation of relative treatment effects on chronic kidney disease between second-line drugs: A sensitivity analysis excluding patients who died

| Treatment | POM | Lower limit | Upper limit | ATE (95% CI) | RR (95% CI) |
| --- | --- | --- | --- | --- | --- |
| ITT |  |  |  |  |  |
| SGLT2i | 0.038 | 0.012 | 0.063 | -0.143 (-0.169, -0.117) | 0.21 (0.07, 0.35) |
| DPP4i | 0.134 | 0.123 | 0.145 | -0.047 (-0.059, -0.035) | 0.74 (0.67, 0.80) |
| TZD | 0.176 | 0.134 | 0.169 | -0.005 (-0.024, 0.013) | 0.97 (0.86, 1.07) |
| SU | 0.181 | 0.158 | 0.193 | 0 | 1 |
| PPA |  |  |  |  |  |
| SGLT2i | 0.024 | 0.002 | 0.046 | -0.179 (-0.202, -0.156) | 0.12 (0.01, 0.23) |
| DPP4i | 0.148 | 0.133 | 0.162 | -0.055 (-0.071, -0.038) | 0.73 (0.65, 0.81) |
| TZD | 0.196 | 0.174 | 0.218 | -0.007 (-0.030, 0.016) | 0.97 (0.85, 1.07) |
| SU | 0.203 | 0.195 | 0.210 | 0 | 1 |
| Modified ITT |  |  |  |  |  |
| SGLT2i | 0.038 | 0.012 | 0.063 | -0.143 (-0.169, -0.117) | 0.21 (0.07, 0.35) |
| DPP4i | 0.134 | 0.123 | 0.145 | -0.047 (-0.059, -0.035) | 0.74 (0.67, 0.80) |
| TZD | 0.176 | 0.134 | 0.169 | -0.005 (-0.024, 0.013) | 0.97 (0.86, 1.07) |
| SU | 0.181 | 0.158 | 0.193 | 0 | 1 |

^ATE: Average treatment effect; CI: Confidence interval; DPP4i: Dipeptidyl peptidase-4 inhibitors; ITT: Intention-to-treat; POM: Potential outcome mean; PPA: Per-protocol analysis; RR: Relative risk; SGLT2i: Sodium-glucose cotransporter-2 inhibitors; SU: Sulfonylureas; TZD: Thiazolidinediones^

**
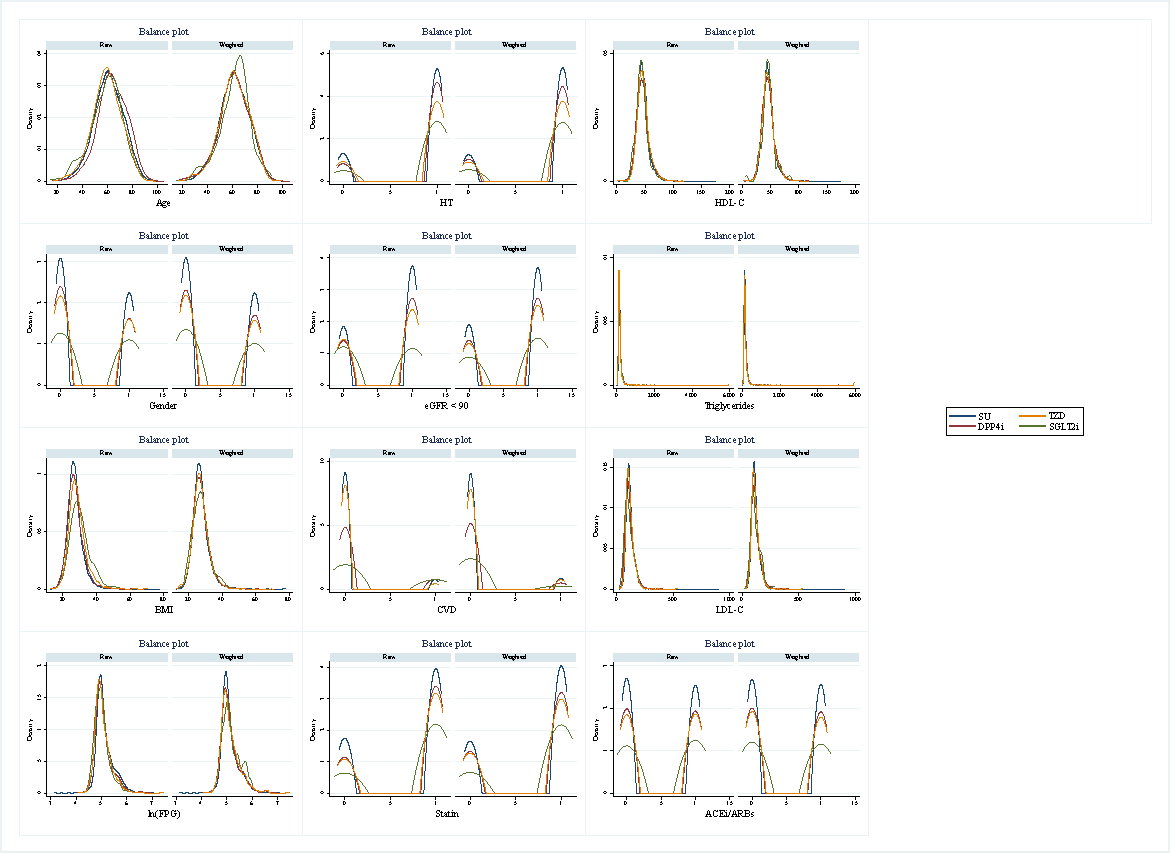
**

Figure S1. Balance plots of variables associated with second-line drug allocation


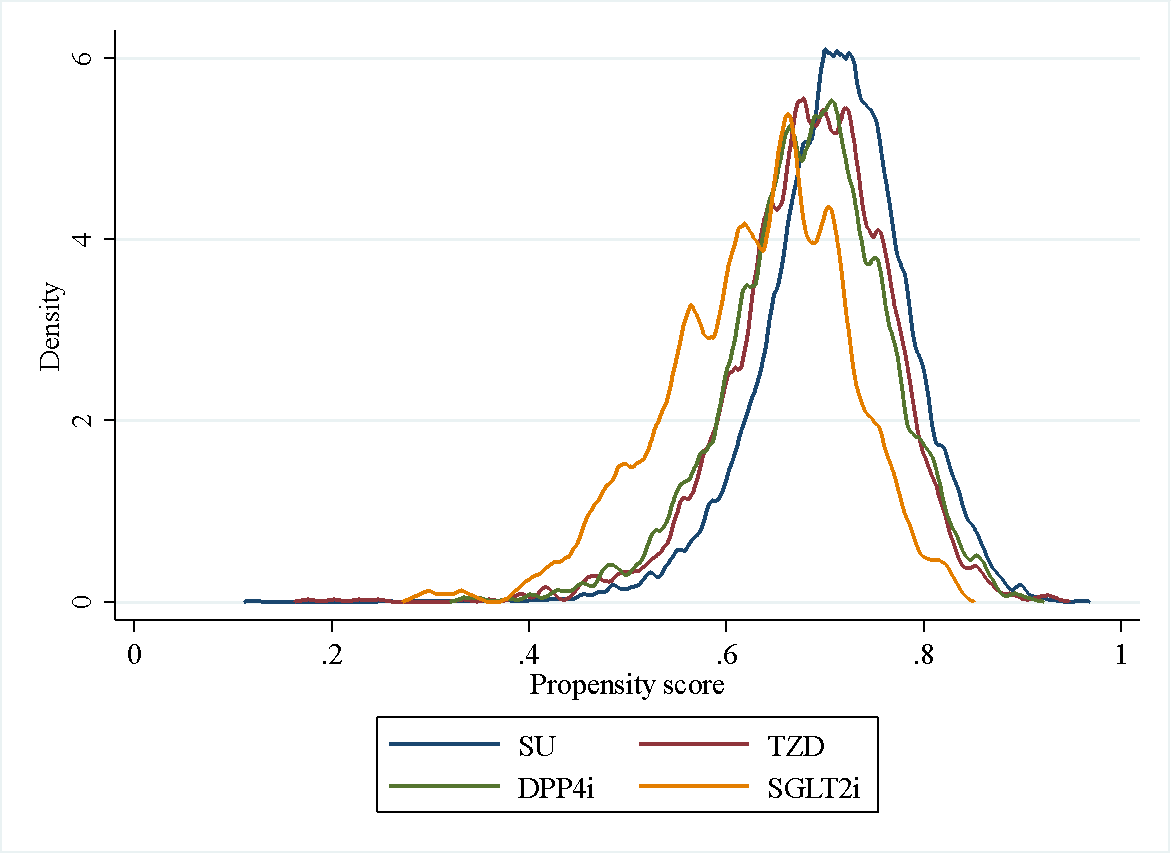


Figure S2. Density plots of the probabilities to receive each second-line drug
